# A contemporary strain of RSV activates primary human monocytes after abortive infection

**DOI:** 10.3389/fimmu.2025.1699818

**Published:** 2025-11-07

**Authors:** Ayse Agac, Martin Ludlow, Marie-Christin Knittler, Chittappen Kandiyil Prajeeth, Giulietta Saletti, Albert D. M. E. Osterhaus, Robert Meineke, Guus F. Rimmelzwaan

**Affiliations:** Research Center for Emerging Infections and Zoonoses, University of Veterinary Medicine Hannover, Hannover, Germany

**Keywords:** monocytes, respiratory syncytial virus (RSV), innate immunity, cytokines and chemokines, activation markers, abortive infection

## Abstract

Respiratory syncytial virus (RSV) is a leading cause of lower respiratory tract infections worldwide, particularly affecting infants, older adults, and immunocompromised individuals. Understanding the cellular immune response to RSV infection is essential for developing effective treatments for infection and its complications. In this study, we investigated the susceptibility of blood-derived primary monocytes and monocytic THP-1 cells to infection with a contemporary RSV A-ON1 strain and characterized the subsequent cytokine and chemokine secretion, as well as the expression of surface markers involved in antigen presentation. Our findings demonstrate that primary monocytes and related THP-1 cells are permissive to abortive infection by RSV, leading to increased expression of proinflammatory cytokines and chemokines, including IP-10, IL-6, and CCL2. Furthermore, primary monocytes expressed CD80, CD86, and HLA-DR upon direct infection or through potential paracrine stimulation. Collectively, these findings demonstrate the activation of monocytes by RSV infection, suggesting their contributory role in orchestrating early immune responses during infection.

## 1 Introduction

Respiratory syncytial virus (RSV) is an enveloped, single-stranded, negative-sense RNA virus belonging to the family *Pneumoviridae* and the genus *Orthopneumovirus*. Two antigenic subgroups (A and B) have been described for RSV (1–3), with genotypes ON-1 (subgroup A) and BA-CC (subgroup B) currently predominant in the human population globally (4, 5).

Infections with RSV are usually asymptomatic or result in mild, flu-like symptoms, including cough, congestion, fever, and fatigue (6). However, infants, immunocompromised individuals, and older adults are at high risk of developing more severe lower respiratory tract disease, characterized by bronchiolitis, pneumonia, croup, rhinorrhea, tachypnea, dyspnea, and a wheezy cough (7, 8). In 2019, more than 33 million cases of RSV-related acute lower respiratory infections (ALRIs) were reported in infants, and approximately 5.2 million cases were reported in older adults worldwide, with approximately 10% leading to hospitalization (9, 10). In older adults, severe infections are often associated with reduced immune system function and immunosenescence (11). In contrast, severe RSV infections in infants are often associated with an imbalanced immune response, characterized by eosinophilia, neutrophilia, and a Th2-type profile (3, 12–14). Furthermore, owing to their anatomical structure, the airways of infants are at increased risk of obstruction by cell debris and mucus (15). These imbalanced immune responses may predispose individuals to developing asthma and wheezing later in life (16–19). The development of countermeasures against RSV infections has been historically challenging, as the first clinical trial in infants with a formalin-inactivated RSV vaccine in the 1950s was halted after two vaccinees died following their first RSV infection (20). Since the early 2000s, virus-neutralizing monoclonal antibodies (Synagis^®^/palivizumab) have been used to protect high-risk infants during their first RSV season (21–23). More recently, monoclonal antibodies, Beyfortus^®^ (nirsevimab) and Enflonsia™ (clesrovimab), were developed against the fusion protein in its prefusion conformation (preF) (24–26); these antibodies have greater neutralizing activity and a longer half-life than palivizumab (27, 28). The stabilization of PreF also led to the first FDA-approved protein-based vaccines for older adults (Abrysvo, Arexvy) and pregnant women (Abrysvo) (29, 30). Furthermore, an mRNA-based vaccine encoding the fusion protein in its prefusion conformation was approved by the FDA for use in older adults and adults at risk for severe infections (31, 32). Although vaccines and monoclonal antibodies have become more readily available in recent years, our understanding of severe RSV infections remains incomplete, particularly regarding the roles of innate and adaptive immune cells in immunopathogenesis. The recent halt of phase I clinical trials in infants vaccinated with Moderna’s PreF mRNA vaccine, which resulted in increased disease severity upon subsequent first infection, underscores the importance of understanding the protective and harmful effects of immunity during RSV infection, which may be crucial in developing safe and effective vaccines for infants (33).

Various immune cells respond to RSV infection with different kinetics (29). However, alveolar macrophages, tissue-resident dendritic cells (DCs), and circulating monocytes are among the first responders to RSV infection and orchestrate immune responses through the secretion of cytokines and chemokines (29). Monocytes, in particular, are recruited during the early stage of infection and respond to MIP-1α, interleukin (IL)-6, IL-8, CCL2, and CCL5, which are secreted by infected epithelial cells and innate immune cells in close vicinity, as demonstrated in murine *in vivo* experiments and human *in vitro* systems (34, 35). In response to *in vitro* infection, monocytes secrete type I interferons (IFNs), tumor necrosis factor-α (TNF-α), granulocyte colony-stimulating factor (G-CSF), and IL-6, enabling the activation and recruitment of innate and adaptive immune cells (36). During infection in infants, monocytes are activated, leading to the upregulation of CD40, CD80, and MHC-I/II, which are involved in antigen presentation (36, 37). Monocytes, therefore, contribute to viral clearance by recruiting and activating immune cells and are involved in initiating adaptive immune responses.

However, immune responses may also contribute to immunopathogenesis, as previous studies have shown that RSV can directly infect immune cells, such as monocytes, macrophages, dendritic cells, or T cells (38–45). Since mononuclear phagocytes are among the first responders to infection, altering their cytokine and chemokine secretion profiles via RSV infection may affect the recruitment and activation of both innate and adaptive immune cells. Upon RSV infection, monocytes isolated from healthy donors exhibit reduced expression of intercellular adhesion molecule-1 and its ligand, thereby affecting the interaction of monocytes with other immune cells (46). Additionally, age-related differences have been described, as monocytes isolated from cord blood are more susceptible to infection than those isolated from adults, suggesting a potential role for monocytes in immunopathogenesis during severe infections in infants (47). Similarly, *in vitro* infection of neonatal alveolar macrophages resulted in impaired IFN-γ production, leading to reduced IFN-γ activation and subsequent immune cell recruitment (46, 48). While the infection of monocytes and alveolar macrophages affects cytokine and chemokine responses, the infection of murine bone marrow-derived DCs impairs the formation of immunological synapses with T cells (49). *In vitro* infection of DCs isolated from human blood further reduces the secretion of type I interferons, ultimately resulting in delayed and deficient activation of T cells and adaptive immune responses with a Th2-type phenotype (49, 50). This Th2-type immune phenotype, characterized by cytokines such as IL-4, IL-5, IL-10, and IL-13, was also observed in severe cases of RSV, which may be attributed to an imbalanced innate immune response (51). Mononuclear phagocytes, therefore, are crucial not only for an early response to infection but also for initiating protective adaptive immune responses. Direct infection of these cells, however, can modulate their response, ultimately resulting in excessive immune cell recruitment and a dysregulated Th2-type immune environment that may underlie severe infections and immunopathology. In the present study, we aimed to investigate the susceptibility and immune activation of primary human monocytes (PMs) in response to infection with a low-passage, contemporary RSV A clinical isolate of the ON1 genotype.

Although data on the role of monocytes and other immune cells during RSV infection are available, these studies were conducted using the laboratory-adapted strains Long and A2, which were isolated in 1956 and 1961, respectively, and have been continuously passaged since then (52, 53). These strains do not accurately reflect currently circulating subtype A strains but are still widely used in research. Molecular differences in viral strains, however, may affect immune cell responses to infections, especially considering that currently circulating RSV strains differ from previous strains in terms of sequence duplication in the second hypervariable domain of the G protein (54, 55), a protein known to be involved in mediating immune evasion and modulation mechanisms (56–58). Differences in innate immune responses elicited by various clinical isolates were also observed upon infection of A549 cells and monocyte-derived macrophages, highlighting the importance of the use of low-passage clinical isolates in research aimed at understanding RSV-mediated immunopathogenesis (59). Compared with the laboratory-adapted A2 strain, the infection of cotton rats and mice with clinical isolates of RSV resulted in increased replication and distinct cell tropism in the upper respiratory tract, indicating that strain-dependent differences in viral dissemination and tropism may be relevant in the context of severe infections (60). Research with contemporary RSV strains and the use of novel approaches are therefore necessary to characterize and understand innate immune responses to infection better, which is fundamental for the development of safe and effective vaccines.

In this study, we investigated the innate immune response of primary monocytes isolated from human blood upon infection with a contemporary strain of the RSV-A ON1 genotype. Using multiparametric flow cytometry and Luminex-based multiplex analysis, we defined the monocyte response to RSV infection in detail. We demonstrate that monocytes are susceptible to infection, resulting in a distinct cytokine and chemokine profile, as well as the upregulation of costimulatory factors involved in antigen presentation. In our study, we included THP-1 cells, an immortalized monocyte-like cell line commonly used as a proxy for PMs. Although THP-1 cells exhibit many monocyte-like features, it is unknown if differences in immune responses to viral infections exist, which may impact the biological relevance of data obtained with THP-1 cells. Comparison of responses of PMs with those of monocytic THP-1 cells revealed substantial differences, indicating that the results obtained with the latter cells should be interpreted with caution. In summary, our data provide new insights into the role of monocytes during RSV infection and provide the foundation for a better understanding of the mechanisms underlying both protective and harmful immune responses to RSV infection.

## 2 Materials and methods

### 2.1 Isolation of peripheral blood mononuclear cells and primary monocytes

Warm buffy coats from anonymous donors were provided by the German Red Cross. Peripheral blood mononuclear cells (PBMCs) were isolated via SepMate tubes and Lymphoprep™ density gradient medium (StemCell Technologies) according to the manufacturer’s instructions. Briefly, donor blood was diluted with an equal volume of wash buffer (Dulbecco’s phosphate-buffered saline [PBS, Capricorn Scientific] supplemented with 2% fetal bovine serum [FBS, Gibco]) and layered onto Lymphoprep™ in a SepMate™ tube (StemCell Technologies). The samples were subsequently centrifuged at 1200 × g for 20 min at room temperature (RT). The PBMC layer was collected and washed twice by centrifugation at 800 × g for 10 min. The cells were treated with ACK lysis buffer (Gibco) for 3 min to remove red blood cell contamination, followed by washing. PBMCs were resuspended in freezing medium (90% FBS+10% DMSO), aliquoted, and cryopreserved at -150°C until further use. Primary monocytes were isolated via negative selection using a classical monocyte isolation kit (Miltenyi Biotec) and an autoMACS Pro Separator (Miltenyi Biotec) according to the manufacturer’s instructions. Isolated monocytes were cultured in R10F (RMPI-1640, 10% FBS, 1% penicillin/streptomycin [P/S], 1% GlutaMAX, 1% nonessential amino acids, 1% sodium-pyruvate, and 1% MEM vitamins [all from Gibco]) and immediately used for infections and downstream analyses.

### 2.2 Cells

THP-1 (TIB-202) and HEp-2 (CCL-23) cells were obtained from the American Type Culture Collection (ATCC, Bethesda, MD). THP-1 cells were cultured in RPMI-1640 supplemented with 10% fetal bovine serum, 1% P/S, and 0.05 mM 2-mercaptoethanol (Gibco) and maintained at a density between 2.5-10 × 10^5^ cells/ml. THP-1 cells were cultured in the absence of differentiating factors to preserve a monocyte-like phenotype. HEp-2 cells were maintained in minimum essential medium (MEM) supplemented with Earl’s salts (MEM-A, Capricorn Scientific), supplemented with 10% FBS and 1% P/S, and passaged at a confluency of 80–90%.

### 2.3 Viruses

Infections were performed via reverse genetics using the RSV-A-0594 strain of the ON1 genotype or a recombinant RSV-A-0594-eGFP strain generated previously (61). Virus stocks were generated by infecting HEp-2 cells with Opti-MEM (Gibco) supplemented with 1% P/S at 60–80% confluency. The virus was harvested upon the appearance of cytopathic effects (3–5 days post-infection) as described previously (56). Briefly, the cells were scraped from the flasks, and the whole-cell suspensions were centrifuged to remove cell debris. The supernatants were mixed with 50% polyethylene glycol (PEG-6000) to a final concentration of 10% and incubated at 4°C for 4 hours (h). Suspensions were centrifuged at 3000 × g for 30 min at 4°C, and pellets containing viral particles were resuspended in Halt’s balanced salt solution (Gibco) containing 20% sucrose. The virus was aliquoted, snap-frozen in liquid nitrogen, and stored at -150°C until further use. Virus titration was performed in HEp-2 cells, and the 50% tissue culture infectious dose (TCID_50_)/mL was determined according to Reed and Muench (62).

### 2.4 Replication kinetics

Primary monocytes and THP-1 cells were infected with RSV-A-0594 or rRSV-A-0594-eGFP to assess viral replication kinetics. HEp-2 cells served as a positive control for productive RSV infection. Virus preparations were diluted in R10F for primary monocytes and THP-1 to prevent loss of viability. HEp-2 cells were infected with RSV diluted in Opti-MEM containing 1% P/S. The cells were inoculated with RSV at a multiplicity of infection (MOI) of 1 and incubated at 37°C and 5% CO_2_ for 2 h. The inocula were then removed, and fresh medium was added to the cells (R10F for monocytes and THP-1 cells, Opti-MEM+1% P/S for HEp-2 cells). Infected cells were incubated at 37°C and 5% CO_2,_ and samples were taken at 0 (after removal of inoculum), 24, 48, and 72 hours post-inoculation (hpi). The cells and supernatants were collected, freeze-thawed in liquid nitrogen twice, and centrifuged at 1000 × g for 10 min at 4°C to remove cell debris. The supernatant was collected, and the viral particles were precipitated with PEG as described above to increase assay sensitivity and to detect changes at low virus titers. Virus preparations obtained from infected HEp-2 cells were processed in the same way to exclude any effects of PEG precipitation on virus infectivity. The virus was then resuspended in HBSS+20% sucrose, snap-frozen in liquid nitrogen, and stored at -150°C until titration on HEp-2 cells was performed. Titers of rRSV-A-0594 were visualized by immunostaining. To this end, the plates were fixed at 5 days post-infection with 4% paraformaldehyde (PFA) and permeabilized with 0.5% Triton X-100. The cells were blocked in PBS containing 5% bovine serum albumin and incubated with polyclonal goat anti-RSV (AB1128, 1:500, Merck). Horseradish peroxidase (HRP)-conjugated donkey anti-goat polyclonal antibody (AB6885, 2 μg/mL; Abcam) was used as the secondary antibody. Staining was visualized using TrueBlue peroxidase substrate (SeraCare). The titers of rRSV-A-0594-eGFP were determined by visualization of eGFP fluorescence using a Leica DM8 fluorescence microscope.

### 2.5 Protein quantification and western blotting

To visualize viral protein translation in infected primary monocytes and THP-1 cells, Western blots targeting the RSV nucleoprotein were performed. To this end, 1 × 10^6^ cells were infected at an MOI of 1, and samples were collected at 0, 24, and 48 hpi. The cells were lysed by resuspending the pellets in M-Per lysis buffer (Thermo Fisher Scientific) containing 1x HALT’s phosphatase and protease inhibitor cocktail (Thermo Fisher Scientific) for 10 min. Cleared lysates were stored at -20°C until further use. The protein concentration was determined by the Quick Start Bradford protein assay (Bio-Rad) according to the manufacturer’s instructions. Five micrograms of total protein were loaded onto a 10% SDS–PAGE gel under reducing conditions with 1x Lane Marker Reducing Sample Buffer (Thermo Fisher Scientific) and subsequently transferred to a Cytiva Amersham™ Hybond™ P 0.45 μm PVDF Membrane (VWR International GmbH). The membranes were blocked with 5% skim milk in Tris-buffered saline containing 0.1% Tween-20 (TBS-T) and probed with rabbit anti-RSV nucleoprotein (Clone HL1246, 0.1 mg/mL, Thermo Fisher Scientific) or mouse anti-ß-actin (BA3R, 0.1 μg/mL, Thermo Fisher Scientific) antibodies overnight at 4°C. The membranes were washed with TBS-T and incubated with an HRP-conjugated goat anti-mouse IgG antibody (A16072, 0.5 μg/mL; Invitrogen) or an HRP-conjugated goat anti-rabbit IgG antibody (ab6721, 0.4 μg/mL; Abcam). The membranes were developed with SuperSignal West Pico PLUS chemiluminescent substrate (Thermo Fisher Scientific) and imaged using a ChemiDoc MP imaging system (Bio-Rad).

### 2.6 RNA isolation and real-time quantitative reverse transcription PCR

For the quantification of cytokine and chemokine gene expression, RNA was isolated from infected monocytes and THP-1 cells. For infection, 1 × 10^6^ cells were infected with RSV-A-0594 at an MOI of 1. The cells and supernatants were collected at 0, 24, and 48 hpi. The samples were subsequently centrifuged at 300 × g for 10 min to separate the cells from the supernatant. The supernatants were collected in separate tubes, and 1x HALT phosphatase and protease inhibitor cocktail was added to prevent protein degradation. The samples were stored at -80°C and used for Luminex multiplex assays (described below). Cellular RNA was isolated using a KingFisher Flex (Thermo Fisher Scientific) and a MagMAX mirVana Total RNA Isolation Kit (Applied Biosystems) according to the manufacturer’s instructions. The RNA concentration was measured by a NanoDrop spectrophotometer (Thermo Fisher Scientific), and the samples were immediately stored at -80°C. RT–qPCR was performed by a SYBR-green-based Luna^®^ Universal One-Step RT–qPCR Kit (New England Biolabs) according to the manufacturer’s instructions. Five nanograms of RNA were used per reaction, and 40 cycles were performed for each run in a LightCycler 96 system (Roche Diagnostics International Ltd.). The data were analyzed using LightCycler 96 SW v1.1.0.1320 software (Roche Diagnostics International Ltd.). Primers were synthesized on the basis of sequences published by OriGene Technologies, whereas primers targeting the RSV-A nucleoprotein were based on (63) (**Supplementary Table 1**).

### 2.7 Luminex multiplex assay

To quantify cytokine and chemokine secretion, a Luminex multiplex assay was performed. The supernatants of the infected cells were collected as described above. A multiplex assay was performed using a 25-plex human cytokine panel (Invitrogen) according to the manufacturer’s instructions. Briefly, 50 μL of cell supernatant was incubated with capture beads, followed by washing and incubation with a biotinylated antibody solution and a streptavidin-RPE solution. The beads were washed again and resuspended in sheath fluid. The beads were acquired by a Luminex™ 200 instrument system (Invitrogen), and the results were analyzed using the ProcartaPlex Analysis app (Invitrogen).

### 2.8 IP-10 enzyme-linked immunosorbent assay

To confirm that the secretion of cytokines and chemokines was due to active viral infections and not a consequence of proteins copurified during virus stock generation, an IP-10 ELISA was performed. The infectious virus or UV-inactivated virus was used to inoculate monocytes and THP-1 cells with rRSV-A-0594 at an MOI of 1. The rRSV-0594 virus was UV-inactivated using a CX-2000 UV Crosslinker (AnalytikJena) at 254 nm with a total fluence of 10,000 mJ/cm². The absence of infectious virus particles in UV-inactivated RSV-A-0594 and rRSV-A-0594-eGFP was confirmed by TCID_50_/ml-based back-titration. The samples were collected at 48 hpi, and the supernatants were separated from the cells by centrifugation at 300 × g for 10 min. The supernatants were supplemented with 1x HALT protease and phosphatase inhibitor and immediately used for IP-10 quantification via ELISA MAX™ Deluxe Set Human CXCL10 (IP-10) (Cat. No. 439904, BioLegend) according to the manufacturer’s instructions.

### 2.9 Multiparametric flow cytometry

Primary monocytes were further characterized by flow cytometric analysis. To this end, 1 × 10^6^ cells were infected with rRSV-A-0594-eGFP or UV-inactivated virus at an MOI of 0.25. Cells treated with lipopolysaccharide (LPS, 100 ng/mL) and IFN-γ (50 ng/mL) served as positive controls for activation. Infected PMs, which were left unstained or stained with isotype control antibodies, served as controls for signal specificity. The cells were harvested via centrifugation at 300 × g for 10 min at 0, 24, and 48 hpi and stained with a LIVE/DEAD™ Fixable Near-IR Dead Cell Stain Kit (Invitrogen). Analyses of cell viability by Live-Dead staining showed high viability of uninfected PMs immediately after isolation (>98%), which significantly decreased upon culturing of cells (38% viability at 48 hours). Flow cytometric analyses of surface marker expression were therefore performed for up to 48 hpi. After Live/Dead staining, cells were incubated with an Fc receptor-blocking reagent (BioLegend), followed by surface staining with monoclonal antibodies directed to and labelled with the following: CD80-BV421 (Clone L307.4, 0.25 μg/mL, Cat. No. 464160, BD Biosciences), CD86-PE-Cy5 (Clone FUN-1, 30 ng/mL, Cat. No. 555659, BD Biosciences), and HLA-DR-PE (Clone L243, 0.2 μg/mL, Cat. No. 307606, BioLegend). Mouse IgG1, k-BV421 (Clone X10, Cat. No. 562438, BD Biosciences), mouse IgG1, k-PE-Cy5 (Clone MOPC-21, Cat. No. 400118, BioLegend), and mouse IgG2a, k-PE (Clone MOPC-173, Cat. No. 400211, BioLegend) were used as isotype controls and at the same concentrations as the respective antibodies. Fc receptor-blocking reagents and antibodies were diluted in BD stain buffer (BD Biosciences). The cells were fixed with Cytofix (BD Biosciences), resuspended in PBS, and acquired using a BD LSR Fortessa X-20 flow cytometer (BD Biosciences) and BD FACSDiva software (version 9.0, BD Biosciences). Analysis was performed using FlowJo software (BD Bioscience, v10.10.0) by gating live, single cells (**Supplementary Figure 3**).

### 2.10 Statistical analysis

To account for inter-donor variability, experiments were performed using multiple (3–4 donors/experiment) donors (the number of donors used is indicated in figure legends; in the figures, each symbol represents results obtained with an individual donor). Statistical analyses were performed using GraphPad Prism v10.4.1. The specific tests used are described in the figure legends. In all analyses, a *p* value of < 0.05 was considered statistically significant (ns = p > 0.05; ∗ = p ≤ 0.05; ∗∗ = p ≤ 0.01; ∗∗∗ = p ≤ 0.001; ∗∗∗∗ = p ≤ 0.0001). For all statistical analyses performed, the normality of the data was assessed using the Shapiro-Wilk test.

## 3 Results

### 3.1 Monocytes are susceptible to RSV infection and support viral protein synthesis

**Figure 1:**
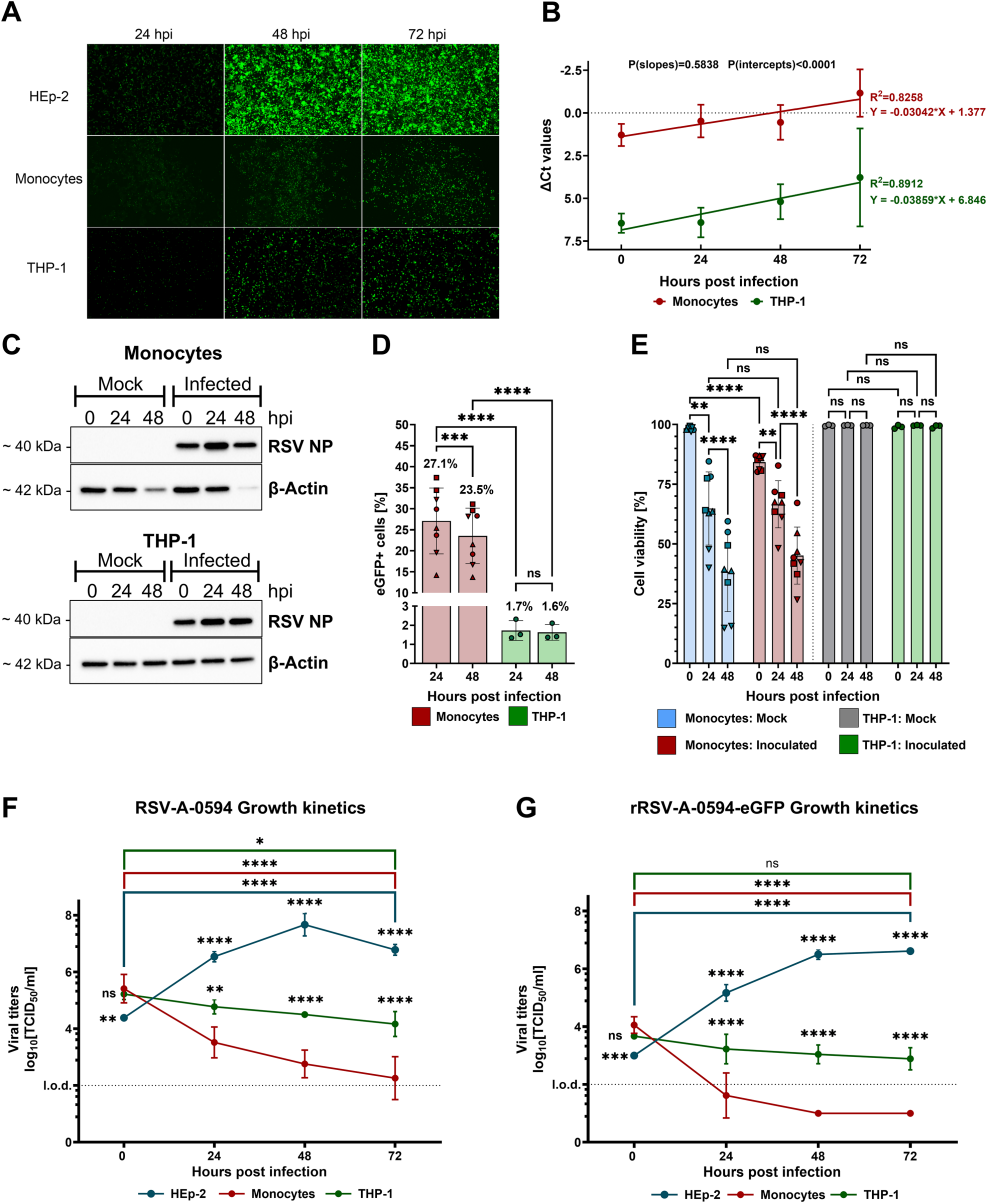
Susceptibility of primary monocytes and THP-1 cells to RSV infection. **(A)** Representative fluorescence images of rRSV-A-0594-eGFP-infected (MOI of 1), Hep-2 (top), primary monocytes (PMs, middle), and THP-1 (bottom) cells. **(B)** RT–qPCR analysis of RSV nucleoprotein (NP) RNA in RSV-A-0594-infected (MOI of 1) PMs (red) and THP-1 cells (green). The ΔCT values are shown (normalized to Phosphoglycerate kinase 1 (PGK-1)), and simple linear regression is applied. P values of the slopes and intercepts are indicated. Data are representative of three independent experiments and donors. **(C)** Western blot analysis of viral NP and β-actin expression in RSV-A-0594-infected (MOI of 1) PMs (top) and THP-1 cells (bottom). Data are representative of three independent experiments and donors. **(D)** Percentages of GFP-positive PMs (red) and THP-1 cells (green) upon rRSV-A-0594-eGFP infection (MOI of 0.25). GFP expression was quantified by flow cytometry. Data are representative of three independent experiments and four different donors. Statistical analysis: two-way ANOVA with Fisher’s LSD test. The shape of the points indicates different donors. **(E)** Percentage of viable cells upon infection of monocytes (red) or THP-1 cells (green) with rRSV-A-0594-eGFP (MOI 0.25). Uninfected PMs (blue) and THP-1 cells (gray) served as controls. Cell viability was assessed by live/dead staining and flow cytometry. Data are representative of three independent experiments and four different donors. The shape of the points indicates different donors. (F+G) Viral replication kinetics of RSV-A-0594 **(F)**- or rRSV-A-0594-eGFP **(G)**-infected (MOI of 1) HEp-2 (blue), PMs (red), and THP-1 cells (green). Data are representative of three independent experiments and donors. The means ± SDs are shown for **(B)**, **(D–G)** Statistical analysis for **(E–G)** two-way ANOVA with Tukey’s *post hoc* test.

To determine whether primary monocytes (PMs) and the related THP-1 cell line are susceptible to infection, we conducted experiments to assess susceptibility, viral protein synthesis, and viral replication kinetics. Permissive HEp-2 cells served as a positive control. The cells were inoculated with rRSV-A-0594-eGFP (MOI of 1) and monitored for eGFP expression for 72 h via fluorescence microscopy. The expression of eGFP was observed in HEp-2, PM, and THP-1 cells at all time points (**Figure 1A**). To quantify viral replication, we assessed the abundance of viral nucleoprotein (NP) transcripts in infected PMs and THP-1 cells by RT–qPCR (**Figure 1B**). The normalized number of viral NP transcripts increased over time in both cell types with comparable kinetics (P[slopes]=0.5838). However, the number of NP transcripts was significantly greater in PMs than in THP-1 cells (P[intercepts]<0.0001). Western blot analysis of viral NPs confirmed these results, revealing that the increase in the number of viral NPs in PMs and THP-1 cells corresponded with the qPCR data (**Figure 1C**). Given the significant differences in NP transcript numbers, we quantified the proportion of rRSV-A-0594-eGFP-infected cells in the respective cell populations infected at an MOI of 0.25 by flow cytometry (**Figure 1D**). At 24 and 48 hpi, 27.1% and 23.5% of the PMs were eGFP+, respectively. For the THP-1 cells, the proportion of eGFP+ cells was much lower, with values of 1.7% and 1.6% at 24 and 48 hpi, respectively. Compared with those of uninfected cells, the effects of RSV on cell viability were assessed by live/dead staining of RSV-inoculated PMs and THP-1 cells (**Figure 1E**). The initial viability of mock-treated PMs was greater than 98%, followed by a significant decrease to 64% at 24 hpi and 38% at 48 hpi. This general loss of viability in uninfected PMs was exacerbated upon infection. The cell viability observed immediately after infection (0 hpi) was 84%, and it declined to 66% and 45% at 24 and 48 hpi, respectively. Apparently, the lifespan of PMs outside the physiological environment is limited, regardless of viral infection. To limit any significant impact of low cell viability on experimental outcomes, experiments were terminated within 72 h after PM isolation. In contrast, uninfected THP-1 cells did not display significant loss of viability during the experiment, with 99% viability. Infection of THP-1 cells did not affect cell viability throughout the experiment. Next, we assessed whether infection was productive by determining infectious virus over a 72-h period. The cells were inoculated with RSV-A-0594 (**Figure 1F**) or rRSV-A-0594-eGFP (**Figure 1G**) at an MOI of 1 to assess potential differences in viral replication between the parental strain and the eGFP-expressing reporter virus. Permissive HEp-2 cells were used as a positive control and showed a significant increase in viral titers, peaking at 48 hpi for both the parental virus and the reporter virus, with titers of 10^8^ TCID_50_/mL and 10^6.7^ TCID_50_/mL, respectively. In contrast, virus titers in THP-1 cells did not increase but decreased significantly after 72 h of infection with RSV-A-0594. Although the viral titer in the rRSV-A-0594-eGFP-infected THP-1 cells decreased from 10^3.7^ TCID_50_/mL at 0 hpi to 10^2.9^ TCID_50_/mL at 72 hpi, this decrease was not statistically significant (p=0.0672). For PMs, a significant reduction in virus titer was observed for both RSV-A-0594 and rRSV-A-0594-eGFP. Notably, the mean rRSV-A-0594-eGFP titer fell below the assay detection limit (10^2^ TCID_50_/mL) as early as 24 hpi. The data show that RSV can successfully enter PMs and support viral protein synthesis, as indicated by eGFP expression in rRSV-A-0594-eGFP-inoculated cells and increased levels of viral NP in Western blots, as well as genome replication, evidenced by higher numbers of NP transcripts. However, production of infectious progeny virus was not observed, suggesting that trafficking of viral proteins or egress of viral particles took place inefficiently, if at all, leading to a gradual decline in viral titers in RSV-infected PMs and THP-1 cells. Collectively, these data show that PMs are more permissive to RSV infection than are THP-1 cells and that infection of PMs and THP-1 cells is abortive.

### 3.2 RSV infection of monocytes induces the gene expression of proinflammatory cytokines and chemokines

**Figure 2:**
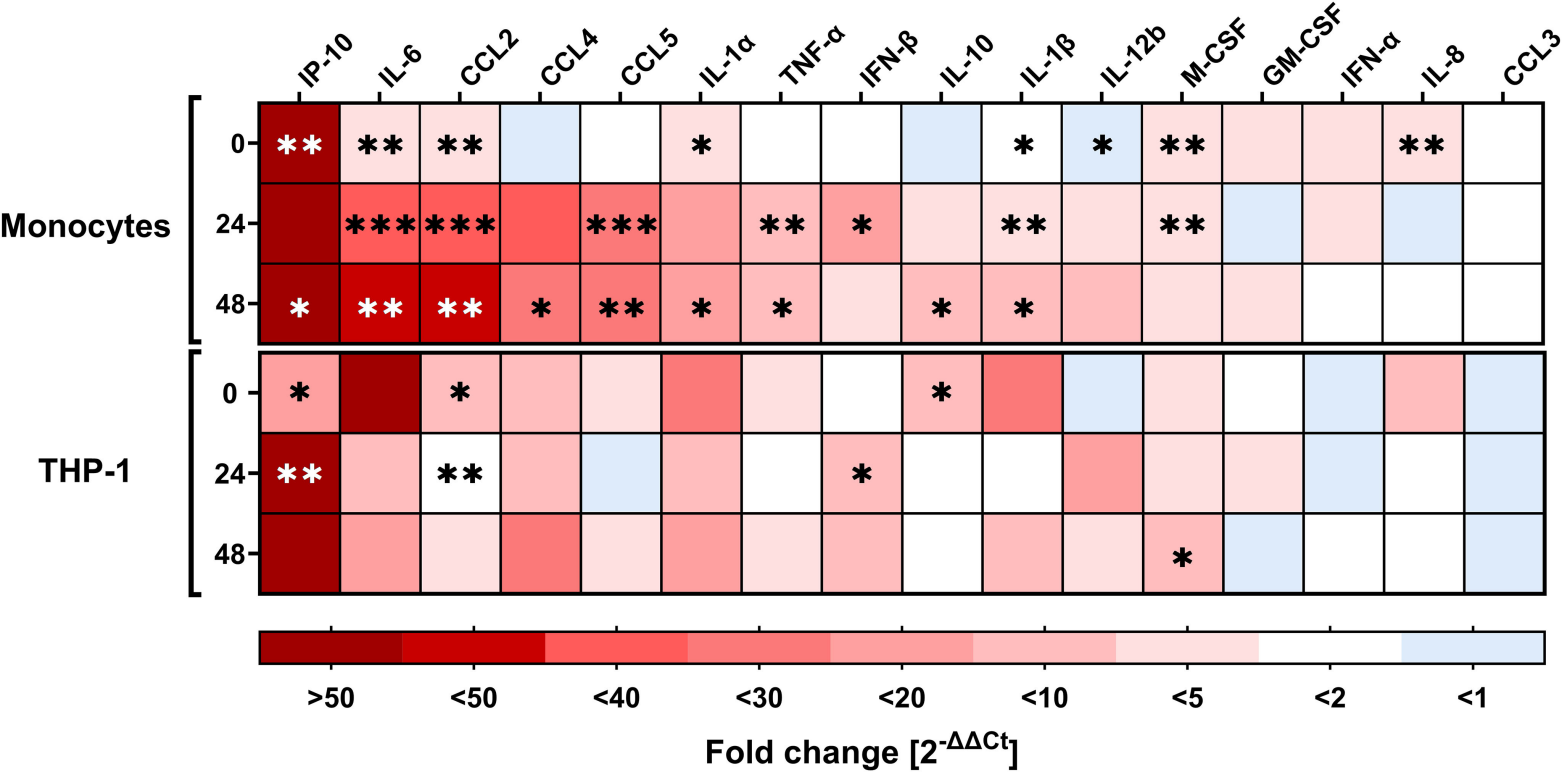
Expression of cytokines and chemokines by monocytes and THP-1 cells upon RSV infection. RT–qPCR analysis of various cytokines/chemokines relevant for immune responses upon viral infection (target genes indicated at the top of the heatmap) in RSV-A-0594-infected (MOI of 1) monocytes (top) and THP-1 cells (bottom) at 0, 24, and 48 hpi. The fold change (2^-ΔΔCT^ values) normalized to that of the housekeeping gene (PGK-1) and mock-infected cells are shown. The means of three independent experiments and donors are shown. Asterisks indicate significant differences between infected and uninfected samples at the indicated time points. The asterisk color varies for better visualization. Statistical analysis: two-way ANOVA with Fisher’s LSD test.

Early innate immune responses to viral infections are characterized by the rapid release of cytokines and chemokines, which serve as the first line of defense and shape subsequent immune responses. We conducted RT–qPCR analysis to quantify the mRNA transcription of cytokines and chemokines involved in viral clearance, immune cell recruitment, and activation in RSV-inoculated PMs and THP-1 cells (**Figure 2**). We observed that IP-10 gene expression was significantly upregulated in infected PMs at 0 and 48 hpi compared with that in uninfected PMs but not at 24 hpi because of increased sample variation (p = 0.1223). PM infection also induced IL-6 and CCL2 gene expression, which peaked at 48 hpi, with a >40-fold increase. RSV infection also induced the expression of other chemokines and cytokines, such as CCL4 (20-fold, peak at 48 hpi), CCL5 (20-fold, peak at 24 hpi), IL-1α (14-fold, peak at 48 hpi), TNF-α (9.7-fold, peak at 48 hpi), IL-1β (6.2-fold, peak at 48 hpi), IFN-β (>10-fold, peak at 24 hpi), and IL-10 (5.5-fold, at 48 hpi). For the other cytokines tested, including GM-CSF, IFN-α, and CCL3, no clear infection-induced transcription kinetics were observed, although a significant increase in expression was observed for IL-8 and M-CSF at 0 hpi. Next, we analyzed infection-induced gene expression in THP-1 cells. Like in PMs, RSV infection induced IP-10 gene expression, which significantly increased at both 0 and 24 hpi. CCL2 expression in infected THP-1 cells was also significantly greater at 0 and 24 hpi, with transcription peaking immediately after infection. In addition to the expression of IFN-β at 24 hpi, that of IL-10 at 0 hpi, and that of M-CSF at 48 hpi, the expression of none of the other tested cytokine or chemokine genes, including IL-6 and CCL4, in RSV-infected THP-1 cells was significantly different from that in uninfected cells, primarily due to greater standard deviations.

### 3.3 Increased secretion of proinflammatory cytokines and chemokines

The observed increase in IP-10 gene expression detected by qPCR in infected monocytes and the important role of IP-10 in the pathogenesis of RSV infection (64–66) indicated that we first assessed IP-10 production by PMs and THP-1 cells via ELISA to test whether its production was dependent on virus replication and not induced by virus particles in trans. Indeed, IP-10 production was induced in PMs and THP-1 cells after stimulation with the infectious virus alone and not after stimulation with the same dose of UV-irradiated virus or in mock-treated cells (**Supplementary Figure 1**).

**Figure 3:**
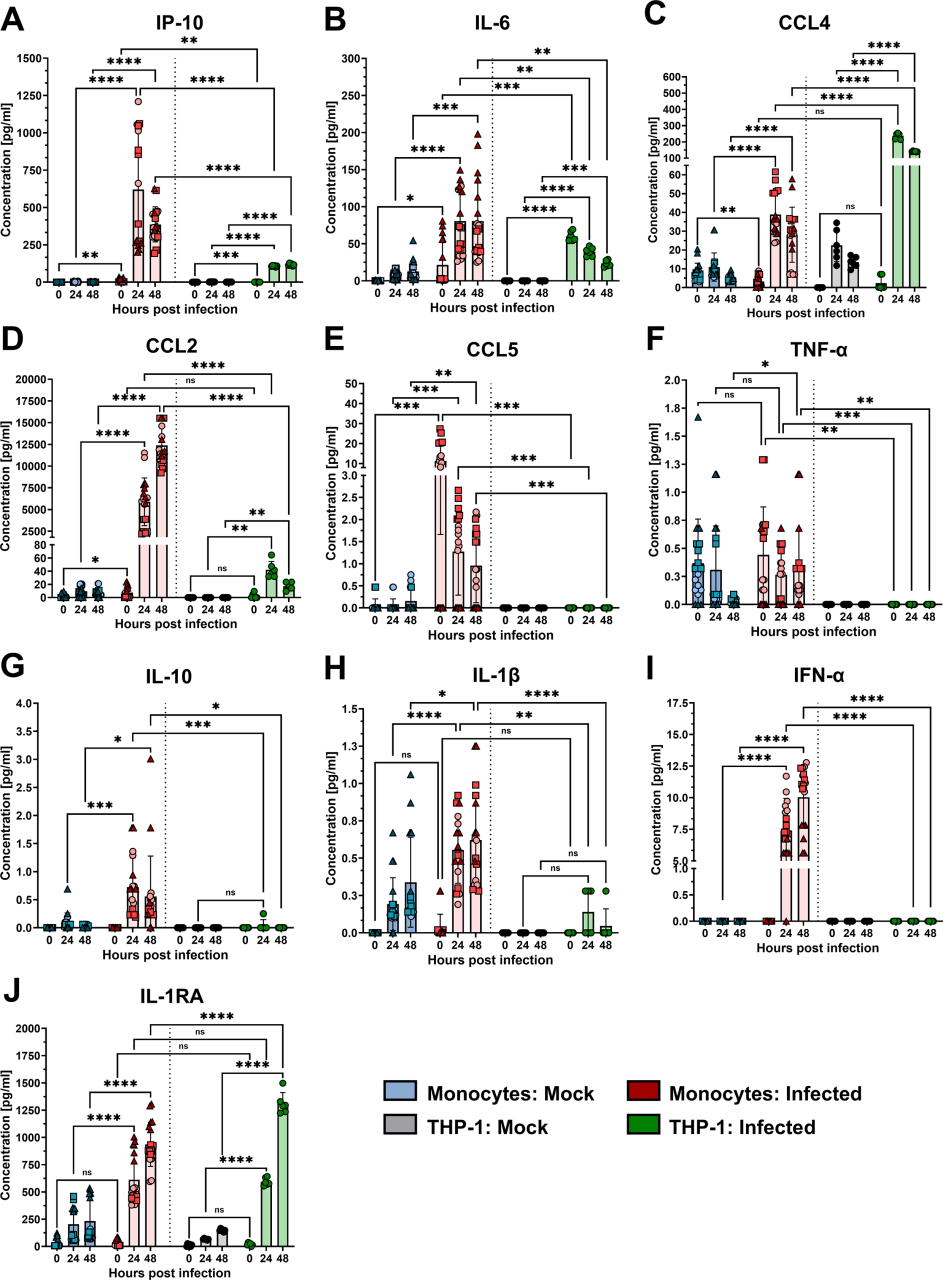
Secretion of cytokines and chemokines from monocytes and THP-1 cells. Luminex multiplex analysis of IP-10 **(A)**, IL-6 **(B)**, CCL4 **(C)**, CCL2 **(D)**, CCL5 **(E)**, TNF-α **(F)**, IL-10 **(G)**, IL-1β **(H)**, IFN-α **(I)**, and IL-1RA **(J)** in supernatants of RSV-A-0594-infected (MOI of 1) monocytes (red) or THP-1 cells (green). Mock-infected monocytes (blue) and THP-1 cells (gray) served as controls. The means ± SDs from three independent experiments and donors are shown. Statistical analysis: two-way ANOVA with Tukey’s *post hoc* test. The shapes of the points indicate different donors. ns (not significant) = p > 0.05; ∗ = p ≤ 0.05; ∗∗ = p ≤ 0.01; ∗∗∗ = p ≤ 0.001; ∗∗∗∗ = p ≤ 0.0001.

To confirm the transcriptomic data, infection-induced cytokine and chemokine production was further tested at the protein level by Luminex multiplex analysis (**Figures 3A-J**). Additionally, Luminex detected the secretion of IP-10 by THP-1 cells and especially PMs (**Figure 3A**), which peaked at 48 hpi (117 pg/mL) and 24 hpi (620 pg/mL), respectively. RSV infection of PMs also induced the production of IL-6, CCL4, CCL2, CCL5, IL-10, IL-1β, IFN-α, and IL-1RA, which are regulators of IL-1 signaling. The TNF-α response was modest in PMs and differed significantly from that in noninfected PMs only at 48 hpi. The secretion of other cytokines and chemokines that were also tested by qPCR is shown in **Supplementary Figure 1**. IL-8 production in RSV-infected PMs did not differ from that in uninfected control cells. CCL3 production also appeared to be greater after infection, but the difference from that in uninfected cells was significant only at 24 hpi. Secretion of GM-CSF and CXCL9 was significantly increased at 24 and 48 hpi, albeit at moderate levels.

Like PMs, THP-1 cells also produced IL-6, CCL4, CCL2, IL-1RA, CCL3, and CXCL9 upon infection with the RSV strain A-0594, although the peak of these responses differed in some cases (**Figure 3**, **Supplementary Figure 2**). In contrast to PMs, we did not detect production of various cytokines and chemokines, including CCL5, TNF-α, IL-10, IL-1β, IFN-α, and GM-CSF, by infected THP-1 cells. Additionally, in contrast to PMs, which fail to produce IL-8 and IL-17A, THP-1 cells produce these cytokines upon RSV infection.

### 3.4 RSV infection induces cell surface marker expression in monocytes

**Figure 4:**
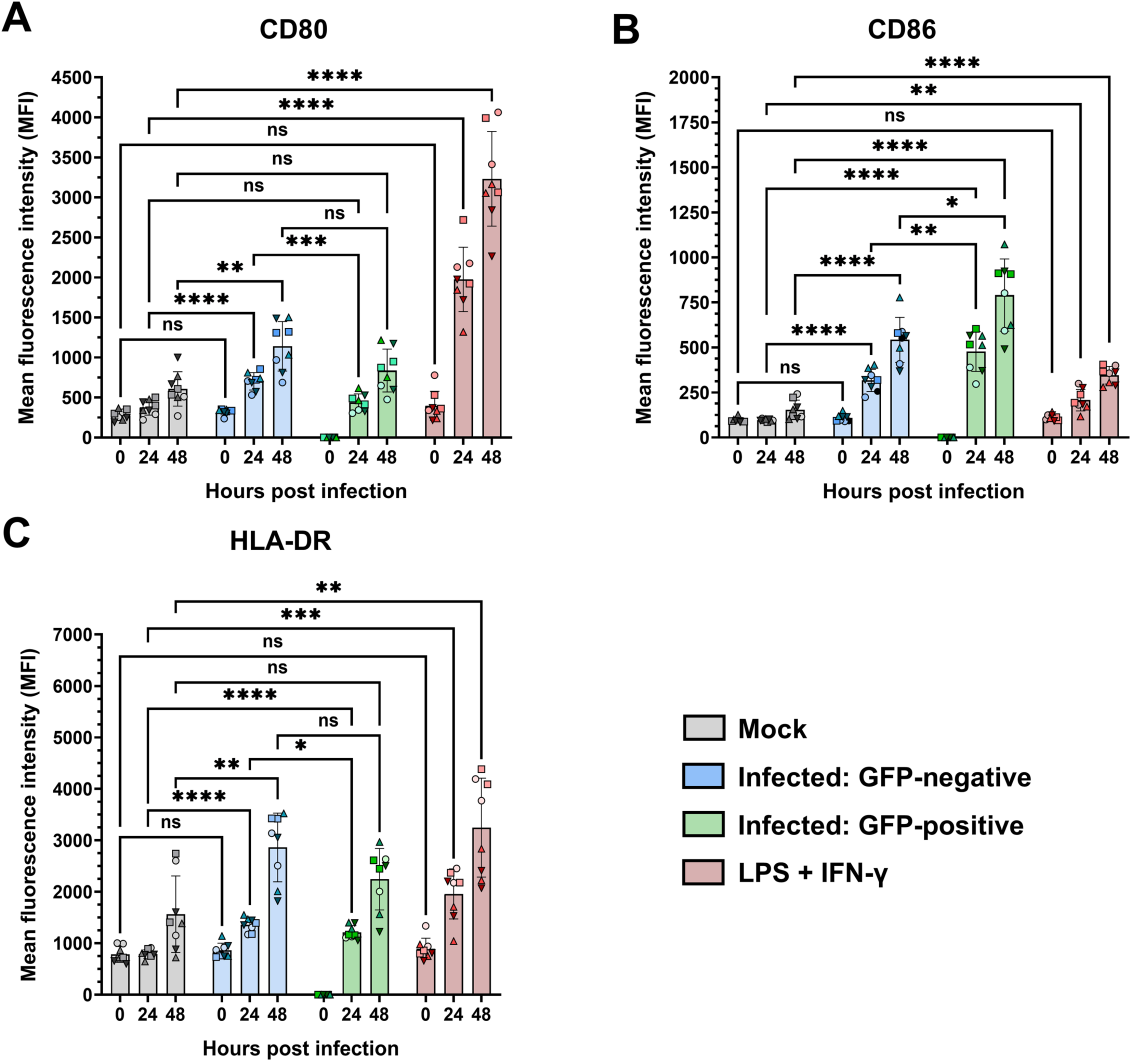
Surface expression of CD80, CD86, and HLA-DR on infected primary monocytes. Flow cytometric analysis of the surface expression of CD80 **(A)**, CD86 **(B)**, and HLA-DR **(C)** on rRSV-A-0594-eGFP-infected (MOI of 0.25) primary monocytes gated on live, single cells. The expression of the targets is shown as the mean fluorescence intensity. RSV-inoculated samples were further divided into GFP-negative (blue) and GFP-positive (green) subpopulations. Mock-treated cells (gray) served as the negative control, while cells treated with LPS (100 ng/ml) and IFN-γ (50 ng/ml) served as the positive control (red). The mean ± SD for two independent experiments and four different donors is shown. Statistical analysis: two-way ANOVA with Fisher’s LSD test. The shapes of the points indicate different donors. ns (not significant) = p > 0.05; ∗ = p ≤ 0.05; ∗∗ = p ≤ 0.01; ∗∗∗ = p ≤ 0.001; ∗∗∗∗ = p ≤ 0.0001.

In addition to the production of cytokines and chemokines, we also assessed the expression of functional surface markers involved in antigen presentation and T-cell activation during RSV infection of monocytes. To this end, PMs were inoculated with rRSV-A-0594-eGFP, and the mean fluorescence intensity of CD80, CD86, and HLA-DR expression was analyzed by flow cytometry. Infection with the reporter virus rRSV-A-0594-eGFP allowed the discrimination of actively infected PMs (eGFP+) and uninfected bystander cells (eGFP-), possibly stimulated in a paracrine manner. Stimulation with UV-inactivated rRSV-A-0594-eGFP served as a control for effects not dependent on infection (**Supplementary Figure 4**). Compared with mock treatment, stimulation with LPS and IFN-γ, which were used as positive controls, induced increased expression of all three markers (CD80, CD86, and HLA-DR) on monocytes at 24 and 48 h poststimulation (**Figure 4**). Upon infection with rRSV-A-0594-eGFP, the expression of CD86, but not that of CD80, was upregulated in eGFP+ PMs (**Figures 4A, B**). The expression of HLA-DR was also increased in these cells at 24 hpi (**Figure 4C**). Interestingly, the expression of CD80, CD86, and HLA-DR (**Figures 4A-C**) was greater in eGFP- PMs than in mock-infected cells, with the highest expression occurring at 48 hpi. While the upregulation of these markers on the surface of eGFP- PMs may result from paracrine stimulation by soluble factors secreted from activated cells, further research is required to confirm this and to identify the responsible cytokines and chemokines. The lower expression of CD80 and HLA-DR on eGFP+ PMs compared to eGFP- bystander PMs at 24 hpi may indicate a possible RSV-mediated immune evasion mechanism. The presence of viral protein in actively infected cells may have interfered with the surface expression of markers involved in antigen presentation. However, further research will be needed to address a potential interference in more detail. Compared with mock treatment, incubation with UV-inactivated virus did not increase the expression of these three surface markers. The specificity of the staining was confirmed by showing that with isotype control antibodies, surface markers were not detected (**Supplementary Figure 4A-C**).

## 4 Discussion

Monocytes play important roles in the innate immune response to viral infections of the respiratory tract and in initiating adaptive immune responses by the secretion of proinflammatory cytokines and chemokines and the activation of virus-specific T cells (29, 67, 68). Therefore, the early responses of these cells contribute to the outcome of RSV infection *in vivo*. However, early monocyte responses may also lead to excessive immune cell infiltration, activation, and inflammation and have been shown to contribute to the pathogenesis of RSV infection (67–70). Severe RSV infections are characterized by excessive Th2-like immune responses associated with the recruitment of eosinophils, mucus hypersecretion, and tissue remodeling, which are all hallmarks of severe airway pathology (12, 13, 71, 72). In the present study, we characterized the response of monocytes, isolated from PBMCs of healthy blood donors, to RSV infection under *in vitro* conditions by investigating their susceptibility to virus infection and subsequent activation. We show that monocytes are susceptible to RSV infection and support viral protein synthesis but do not produce infectious viral progeny. This finding contrasts with those of previous studies, which revealed productive infection of human blood-derived monocytes and monocyte-derived cells (38, 40, 47). An important difference between our study and previous studies is that we used a contemporary RSV A strain of the ON1 genotype (61), whereas the other studies used laboratory-adapted strains, which may possess different properties.

This abortive infection induces the activation of monocytes, which subsequently produce various proinflammatory cytokines and chemokines and express MHC class II and the costimulatory molecules CD80 and CD86, which are important for T-cell activation. More specifically, we showed by qPCR and Luminex multiplex assay that RSV infection of monocytes induced the expression and secretion of cytokines and chemokines involved in viral clearance and immune cell recruitment, such as IP-10, IL-6, and CCL2. Although qPCR revealed the expression of genes encoding the cytokines IL-1α, TNF-α, IL-1β, IL-10, and IFN-β and the chemokines M-CSF, CCL4, and CCL5, their secretion by RSV-infected monocytes was relatively modest. These data suggest that upon RSV infection, monocytes can regulate cytokine production in a posttranscriptional or posttranslational manner to prevent hyperinflammation (73, 74). Furthermore, upon RSV infection, monocytes produced increased levels of IL-1RA, an antagonist of IL-1 signaling, indicating that RSV infection induced both proinflammatory and anti-inflammatory immune responses in monocytes. Monitoring monocyte responses over an extended time period post-inoculation enabled assessment of the kinetics of cytokine and chemokine production. The immediate response to RSV infection is characterized by the secretion of the proinflammatory chemokine IP-10, which is induced by type I and II interferons and a chemoattractant for mainly T cells and natural killer cells (75, 76). The secretion of proinflammatory cytokines, such as IL-6, IL-1β, and IFN-α, displayed a more sustained pattern. The same trend was observed for CCL2 levels, indicating that RSV-infected monocytes can secrete cytokines and chemokines for extended periods, driving inflammation that may contribute to the pathogenesis of RSV infection (77–82). As indicated above, the production of anti-inflammatory molecules, such as IL-1RA, may be important for counteracting inflammatory responses and preventing tissue damage (83). Our findings are in agreement with those of other studies showing the production of the proinflammatory mediators IL-6, IP-10, and CCL2, as well as the anti-inflammatory cytokine IL-1RA, by RSV-infected monocytes (84, 85). Interestingly, the production of IL-6, IL-8, IL-10, and IFN-α has been shown to be correlated with disease severity in RSV-infected children (86–88). In contrast, increased IP-10 levels in the nasal epithelium of infected children are inversely correlated with disease severity, demonstrating the antiviral properties of IP-10 (89). In contrast to previous studies, our study revealed only moderate production of TNF-α, IFN-α/β, IL-10, and IL-1β (84, 85, 90–92). Several factors may underlie this discrepancy, including the RSV strain used, multiplicity of infection, and variation in the study subjects.

The implications of our findings obtained with primary monocytes *in vitro* for the *in vivo* situation and pathogenesis of RSV infection is not entirely clear. Recently, a correlation between RSV disease severity and various inflammatory markers in infants has been implicated, including prolonged hospitalization with a lower monocyte-to-lymphocyte ratio, indicating that monocytes contribute to protective immune responses (93). Abortive infection of primary monocytes may implicate their role as a ‘sink hole’ for virus particles, therefore reducing virus dissemination in infected individuals. In severely infected children, increased levels of monocyte-derived IL-10 during the convalescent phase were primarily associated with recurrent wheezing (94). The cytokine expression profile of RSV-infected primary monocytes observed in the present study indicates that monocytes contribute to an early pro-inflammatory response, through the secretion of IP-10, IL-6, and CCL2. The cytokine expression profile of monocytes in the two extremes of the age spectrum may differ from that in healthy adult subjects that we used in our studies, which, in part, may explain the susceptibilities to RSV infections of these age groups. Further *in vivo* studies are required to address these differences.

We also demonstrated the RSV infection-induced activation of monocytes via the expression of CD80, CD86, and HLA-DR on the cell surface upon infection. The use of the eGFP-reporter virus allowed us to analyze cells that were actively infected (eGFP+) or not (eGFP-). Compared with mock-infected cells, both cell populations expressed these three surface markers, indicating that the eGFP- cells may have been activated by paracrine stimulation. Interestingly, the expression of CD80 and HLA-DR, but not that of CD86, was lower in eGFP+ cells than in eGFP- cells. The underlying mechanism and the biological relevance of these findings are poorly understood and require further investigation. It is possible that infected monocytes have a reduced capacity to present antigens and activate virus-specific T cells. Previous studies have shown that monocytes isolated from RSV-infected infants exhibit reduced HLA-DR expression and IL-10 secretion, which correlates with disease severity (37). Reduced antigen presentation during RSV infection has also been demonstrated in monocyte-derived dendritic cells (42). Although infection induced CD80, CD83, CD86, and HLA class II expression on the surface of DCs, the activation of CD4+ T cells was impaired, which is consistent with findings in RSV-infected murine DCs, where the formation of immunological synapses was impaired (41, 42, 49, 95). The higher CD86 expression in the eGFP+ PMs may reflect compensation for the reduced CD80 expression. Although both CD80 and CD86 provide costimulatory signals through CD28, it remains unclear whether they can fully compensate for each other’s functions during viral infections (96). Further T cell stimulation studies are required to confirm any possible immune modulatory effects during RSV infection on the antigen-presenting capacity of monocytes.

Because monocytic THP-1 cells are frequently used as proxies for primary monocytes (also in the context of RSV) (56, 97, 98), we compared their response to RSV infection with that of PMs. Our findings revealed substantial differences in the immune response to RSV infection between PMs and THP-1 cells. Although they are susceptible to infection, the percentage of infected THP-1 cells was ten times lower than that of PMs. RSV infection triggered cytokine and chemokine responses in THP-1 cells similar to those in PMs. However, the secretion of key cytokines and chemokines differed significantly. IP-10, IL-6, and CCL2, which are secreted by monocytes, are also secreted by THP-1 cells, albeit to a lesser extent. Other important cytokines and chemokines, such as CCL5, TNF-α, IL-10, IL-1β, and IFN-α, were not detectable in the supernatants of infected THP-1 cells at any time point, although a slight increase in transcription was observed. A previous study comparing the responses of THP-1 cells and PMs after RSV infection reported comparable results for both cell types, although the cytokine secretion levels were generally lower in THP-1 cells than in control cells (84). This study used the laboratory-adapted RSV strain Long, which may have accounted for the generally high cytokine responses observed in THP-1 cells compared with our findings with the clinical isolate RSV-A-0549. As previously shown, the infectability of THP-1 cells is strain dependent (99), and our data indicate that the contemporary RSV-A-0549 strain infects THP-1 cells inefficiently and fails to induce the secretion of key cytokines and chemokines. Based on our findings, responses to RSV infection observed with THP-1 cells, as proxy for primary monocytes, should be interpreted with caution.

Although our study indicates that abortive infection of monocytes may play an important role during RSV infection, it is unclear to what extent it contributes to the pathogenesis of RSV infection, especially in patients at high risk for RSV infection. The use of monocytes from infants or older adults may provide further insights into the role of monocytes during severe RSV infections. Another limitation of our study is the *in vitro* culture conditions of the isolated monocytes. Clearly, the microenvironment *in vivo* is more complex and may influence the behavior of monocytes at the site of infection. Further studies are needed to address these limitations, for example, by using precision-cut lung slices, organoid models of the lung, or conducting *in vivo* experiments. Finally, we used an isolate of the RSV-A-ON1 genotype obtained from a patient. It is unclear how infection with viruses of other genotypes, especially those from the RSV-B subgroup, affects monocyte activity. Although recent studies suggest that there are no differences in disease severity between RSV A and RSV B (100, 101), further research is needed to obtain a better understanding of the infection of monocytes by RSV.

In summary, we characterized the infection of primary human monocytes with the contemporary RSV strain A-0549 and the subsequent immune response in these cells. The use of a contemporary RSV strain most likely better reflects current RSV infections than the commonly used laboratory-adapted strains, such as Long and A2. Our findings show that monocytes can become abortively infected, leading to their activation and the production of various proinflammatory and anti-inflammatory cytokines and chemokines during the early stages of infection. As monocytes are among the earliest responders to RSV infection and contribute to early inflammatory processes and immune cell recruitment (70, 102), they play a decisive role in the outcome of respiratory infections (68, 103, 104). We further demonstrated that the susceptibility of commonly used THP-1 cells to infection and the subsequent immune response differ from those of monocytes. Overall, the present study advances our understanding of virus–host interactions and the potential role of monocytes during RSV infection, providing a critical foundation for re-evaluating innate immune activation mechanisms and developing immunomodulatory strategies against severe RSV disease. Further studies with more complex *in vitro* or *in vivo* systems will provide further insights into the role of (infected) monocytes in the pathogenesis of RSV infections.

## Data Availability

The raw data supporting the conclusions of this article will be made available by the authors, without undue reservation.

## Supplementary material

Supplementary Figure 1 Secretion of IP-10 from monocytes and THP-1 cells. IP-10 ELISA of supernatants from mock-treated, UV-inactivated virus-inoculated, or live virus-inoculated (MOI of 1) PMs and THP-1 cells at 48 hpi. The means ± SDs from two independent experiments and three different donors are shown. Statistical analysis: ordinary one-way ANOVA with Fisher’s LSD test. The shapes of the points indicate different donors.

Supplementary Figure 2 Secretion of cytokines and chemokines from monocytes and THP-1 cells. **(A-F)** Luminex multiplex analysis of IL-8 **(A)**, IL-12 **(B)**, CCL3 **(C)**, GM-CSF **(D)**, CXCL9 **(E),** and IL-17A **(F)** in supernatants of RSV-A-0594-inoculated (MOI of 1) monocytes (red) or THP-1 cells (green). Mock-treated monocytes (blue) and THP-1 cells (gray) served as controls. The means ± SDs from three independent experiments and donors are shown. Statistical analysis: two-way ANOVA with Tukey’s *post hoc* test. The shapes of the points indicate different donors.

Supplementary Figure 3 Gating strategy for flow cytometric analysis. Monocytes were acquired by flow cytometry and gated on single, live cells for analysis. The mean fluorescence intensity (MFI) was calculated for the eGFP-negative population only in the controls, whereas the MFI was calculated for both the eGFP-positive and eGFP-negative populations in the RSV-inoculated samples.

Supplementary Figure 4 Surface expression of CD80, CD86, and HLA-DR on infected primary monocytes. Flow cytometric analysis of the surface expression of CD80 **(A)**, CD86 **(B)**, and HLA-DR **(C)** on the surface of primary monocytes gated on live, single cells. Live virus-inoculated samples were further divided into GFP-negative (blue) and GFP-positive (green) subpopulations. Mock-treated cells (gray), unstained, inoculated cells (light pink, magenta), inoculated cells stained with isotype controls (yellow, orange), and cells inoculated with UV-inactivated virus (lilac) served as controls. The mean ± SD for two independent experiments and four different donors is shown. Statistical analysis: two-way ANOVA with Fisher’s LSD test. A comparison of the controls with the mock-treated and infected subpopulations is shown (only significant differences are shown). The shapes of the points indicate different donors.
