# Supplementary material for: A contemporary strain of RSV activates primary human monocytes after abortive infection
Source: Front Immunol. 2025 Nov 7;16:1699818. doi: 10.3389/fimmu.2025.1699818 (PMC12634557; doi:10.3389/fimmu.2025.1699818)
Supplement: Supplementary Figure 1 — Secretion of IP-10 from monocytes and THP-1 cells. IP-10 ELISA of supernatants from mock-treated, UV-inactivated virus-inoculated, or live virus-inoculated (MOI of 1) PMs and THP-1 cells at 48 hpi. The means ± SDs from two independent experiments and three different donors are shown. Statistical analysis: ordinary one-way ANOVA with Fisher’s LSD test. The shapes of the points indicate different donors. [file DataSheet1.docx]

**
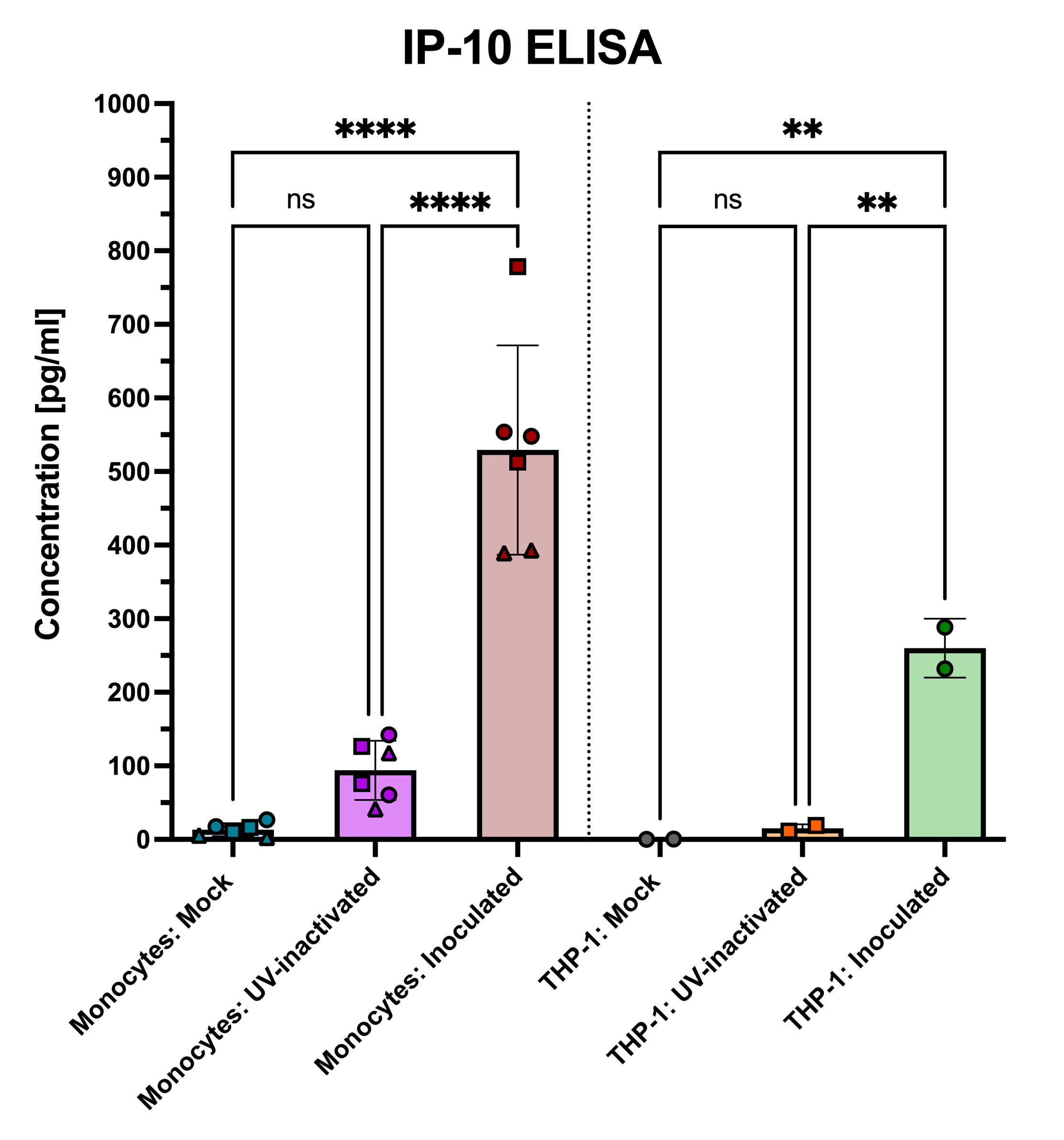
Supplemental Figure 1:**

**
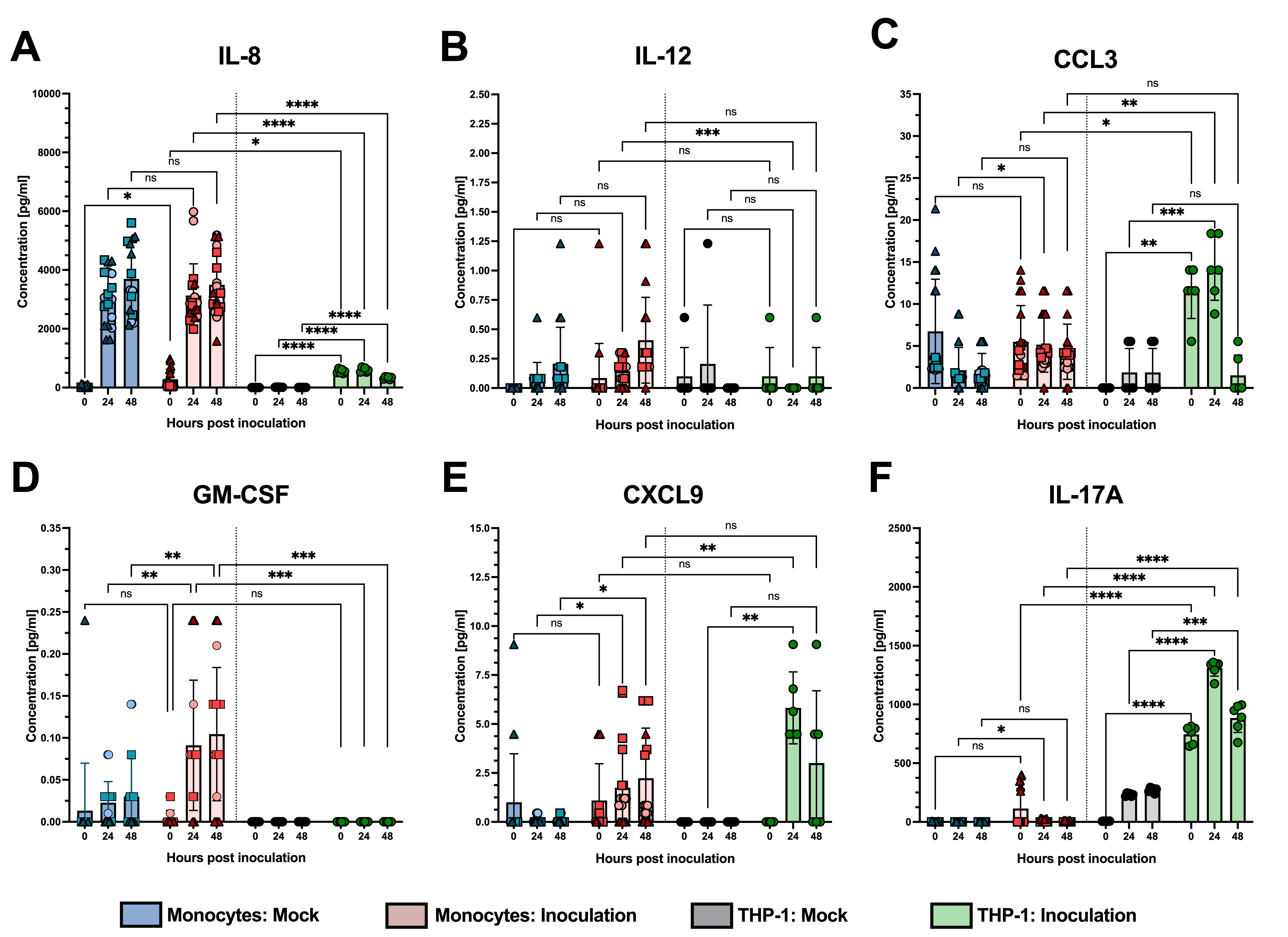
Supplemental Figure 2:**

**Supplemental Figure 3:**


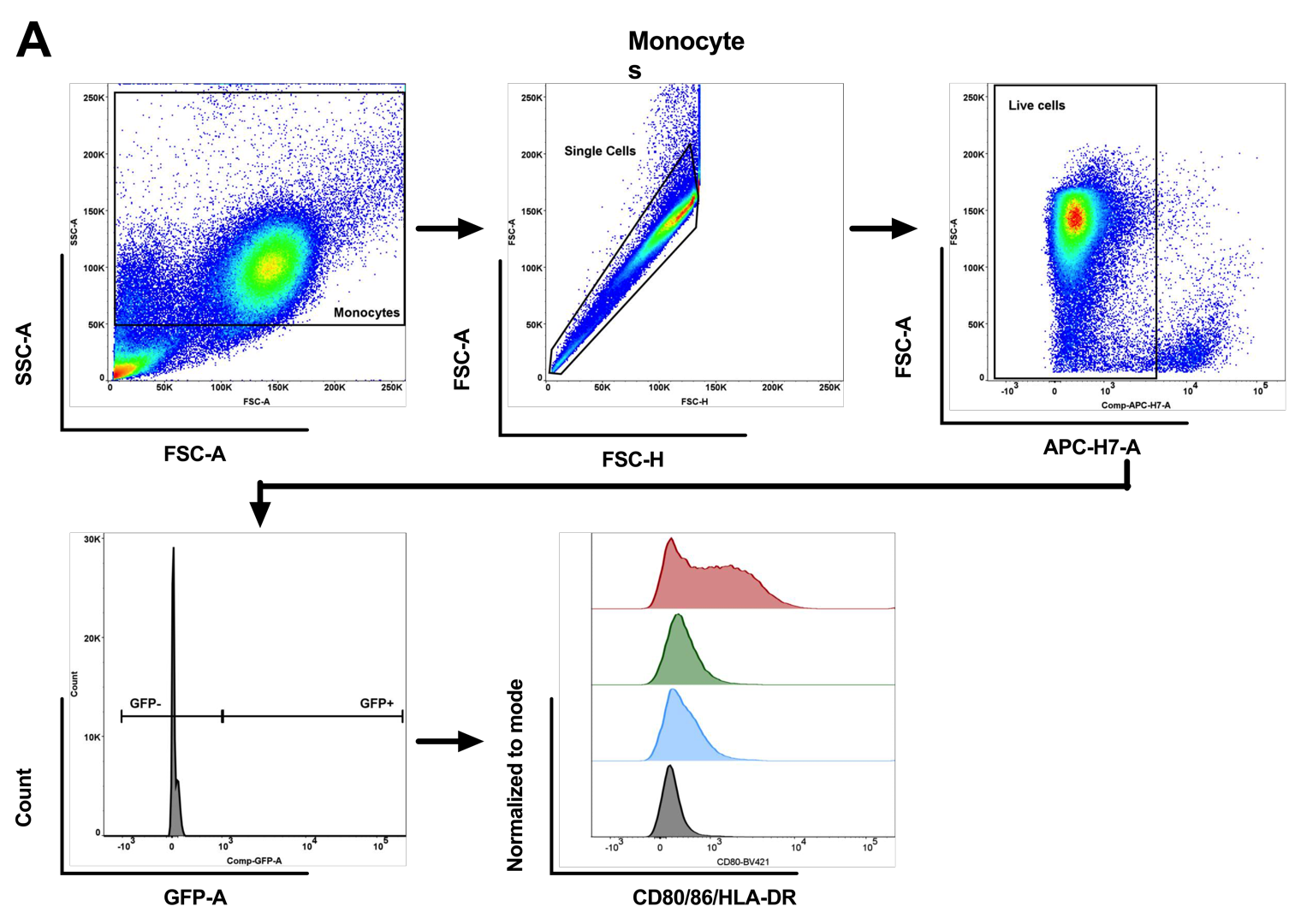


**
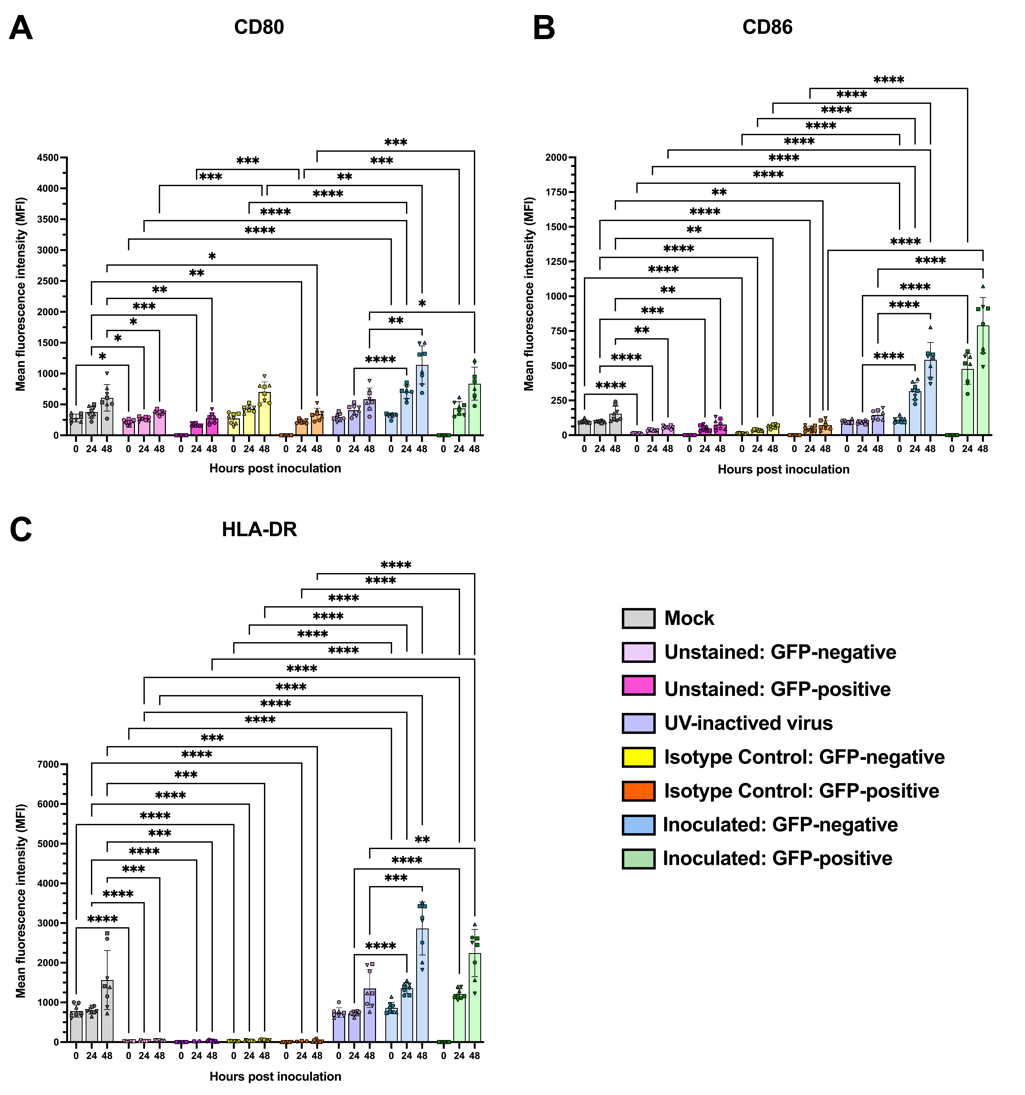
Supplemental Figure 4:**

**Supplemental Table 1: Summary of RT-qPCR target sequences and Tm temperature**
